# Postnatal Care: Levels and Determinants in Morocco

**Published:** 2017-02

**Authors:** Noureddine ELKHOUDRI, Abdellatif BAALI, Hakima AMOR

**Affiliations:** 1. Laboratory of Science and Health Technologies, Higher Institute of Health Sciences University Hassan First Settat, Marrakech, Morocco; 2. Dept. of Biology, Laboratory of Human Ecology, Semlalia School of Sciences, Cadi Ayyad University, Marrakech, Morocco

**Keywords:** Determinants, Postnatal consultation use, Morocco, Women

## Abstract

**Background:**

Despite the importance of the postnatal consultation, in Morocco, only 22% of women attended these consultations. The aim of this study was to identify associated factors with these consultations and offer suggestions to improve their use.

**Methods:**

This study was conducted in 2014 in Marrakech. A sample of women in reproductive age (15–49 yr) giving birth during 2013 year was enrolled. They were examined in the public health centers for postnatal consultation or for the BCG. A descriptive and analytic cross-sectional survey was conducted. All participants (n=1029) provided consent before participating in the survey. A questionnaire makes it possible to collect information about socio-demographic, knowledge and perception of women regarding these consultations.

**Results:**

The proportion of women who attended a postnatal consultation was 30.1%. Lack of information (87%), lack of complications (68.6%); health professional poor reception (42%) and financial difficulties (3.3%) were the main reasons that hinder these consultations. In addition, women of rural origin, low education level, and low socioeconomic status are important determinants associated with non-use of postnatal consultation.

**Conclusion:**

This study confirmed the low rate of these consultations. Various determinants explain this fact. The fight against illiteracy, improving household living standards, sensitization of women on the importance of postpartum care, awareness and capacity building of health professionals in the postnatal consultation and communication, and the development of a system of home visits for non-users of postnatal care allow improving the postnatal consultation rate.

## Introduction

Maternal and child health is at the heart of the Millennium Development Goals (MDO 4 and 5). Every year worldwide, about 350000 women die in childbirth and about 8 million children die before their fifth birthday, more than 99% of these mortalities occur in developing countries (1).

The period of the first six weeks of postnatal is a risky period whether any consultations for mothers and their newborns were not provided (2). Two-thirds of maternal and neonatal deaths occur after childbirth (3, 4). Postnatal consultation (PNC) remains one of the most important means to reduce maternal and neonatal mortality.

In Morocco, the couple newborn mother should benefit from three systematic postnatal consultations (after delivery, the 8th day after delivery and the 40th day after delivery) (5). However, despite efforts made by the Ministry of Health, the results fall short. In fact, the last National Survey on Population and Family Health (6) shows that only 22% of women who received PNC, even if these medical services are provided without charge by the public health centers. In addition, inequalities persist in access to these consultations according to areas (urban and rural) and socioeconomic levels.

The consequences of such a situation are failures in the health of mothers and children. This not only maintains high rates of maternal and neonatal morbid-mortality but also spending on maternal and child health programs.

We posed the following research question: what are the determinants that influence the PNC use? Therefore, we conducted this study in order to identify the factors associated with the PNC use in the city of Marrakesh and offer suggestions to improve their use.

## Methods

### Study Site

This study was conducted in the city of Marrakech, Morocco. It is situated in the center of Morocco and has a population of 1148000 habitats. Maternal health services in the city are offered by sixty-three public health centers with six public ‘delivery houses’ (peripheral primary level), one district hospital, one university hospital, eight private clinics, thirty-four private general physicians and thirteen private laboratories (7).

### Study population

A sample of women in reproductive age (15–49 yr) giving birth during the year preceding the survey was enrolled. They visited the public health centers in the city of Marrakech, Morocco for PNC or for BCG vaccination for their newborns, during the data collection period (Jan–Oct 2014). These women were randomly enrolled and 93% of eligible patients agreed to participate in the study.

### Study design

This was a descriptive and analytic cross-sectional survey. The cluster random sampling was used to select six health centers from the list of these centers in Marrakesh. Subsequently, 172 women were included from the patients examined in each selected health center, giving 1029 women. All participants provided consent before participating in the survey. The information was collected anonymously and confidentially.

A pretested questionnaire makes it possible to collect socio-demographic and health information that might be associated with the PNC use, and knowledge and perception of women regarding PNC. The independent variables used in this study were origin and age of women, education level, couple’s occupations, parity, failed pregnancy and the place of birth. Dependent variable was the PNC use.

### Data analysis

A descriptive analysis was performed using means, standard deviations (SD) and proportions as appropriate. To estimate the significance of the differences observed between the means, the Chi-square test was used for categorical variables. The multivariate analysis which allows the elimination of the confounding factors and entering the weight of the associated variables with the PNC use in the bivariate analysis (*P*<0.2), was used to identify factors independently associated with the PNC use. Associations were measured in Odds ratio (OR) with 95% confidence intervals (95% CI). The statistical significance was set at *P*<0.05 and SPSS ver. 10.1 (Chicago, IL, USA) was used for all statistical analysis.

## Results

### Socio-demographic and health characteristics of studied women

Women’s age ranged from 15 to 49 yr, with an average of 28.2 (SD=6.4). All participants were married, and those aged over 35 in their latest childbirth represent 13.9%.

Rate illiteracy among women was 25.1%, twenty-nine percent attended primary-school level, 37.3% secondary-school level, and only 8.6% reached higher levels in their studies. For the spouses, illiteracy rate was 15%, with 32.1% reaching primary level, 37.7% secondary level and 15.3% reaching higher levels of studies.

Most of the participants were not employed (91.5%) and only 28.6% had medical insurance.

All spouses were employed, with 74.1% being workers, craftsmen, employees, drivers, shopkeepers etc. (grouped in the socio-professional category 1: SPC1), and 25.9% were either state functionaries or had liberal professions (grouped in the socio-professional category 2: SPC2).

70.8% of the participants have not attended antenatal consultation, and the majority of them (97.6%) delivered in a health facility, with 81% in public hospitals and 16.7% in private clinics. Fifteen percent of the enrolled women had caesarean delivery.

### Postnatal consultation

**Fig. 1:**
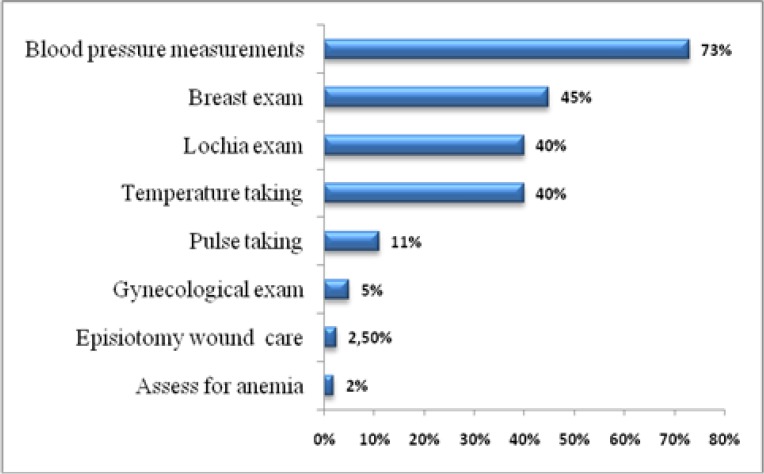
Details of the provided exams during post-natal consultations

The proportion of women who attended a postnatal consultation was 30.1%. Fig. 1 shows the details of the provided exams during these consultations. The blood pressure measurement was performed in 73% of women; this exam confirms the return of blood pressure normal values. The breast exam was performed for 45% of women. Temperature taking and lochia exam were performed for 40% of women. The pulse-taking exam was done for 11% of women. Finally, gynecological exam was done only for 5% of women. Almost all of the respondents (97%) visited the health centers for the PNC and/or BCG for their newborns eight days after birth, the average was 18 d [min=4 d; max=51 d].

### Determinants of postnatal consultation use

According to women’s declarations, the following were the main reasons to hinder PNC: lack of information (87%), lack of complications (68.6%), health professional poor reception (42%) and financial difficulties (3.3%).

Bivariate analysis shows correlations between the PNC use and the studied variables Table 1.

**Table 1:** Associations between the studied variables and the PNC use

**Variables**	**Modalities**	**Postnatal consultation**				**Test** χ**2**

		**No**		**Yes**		
		**%**	**n**	**%**	**n**	
Woman’s age (year)	< 35	70.4	541	29.6	227	0.46 ns
	≥ 35	68.2	178	31.8	83	
Woman’s origin	Rural	76.5	367	23.5	113	18.5^***^
	Urban	64.1	352	35.9	197	
Woman’s education level	Illiterate	77.9	201	22.1	57	30.6^***^
	Primary	76.5	228	23.5	70	
	Secondary and more	61.3	290	38.7	183	
Spouse’s education level	Illiterate	81.2	125	18.8	29	40.9^***^
	Primary	78.8	260	21.2	70	
	Secondary and more	61.3	334	38.7	211	
Woman’s employment	Active	45.7	37	54.3	44	24.4^***^
	Inactive	71,9	682	28,1	266	
Spouse’s SPC	SPC1	72,8	646	27,2	241	26.7^***^
	SPC2	51,4	73	48,6	69	
Parity	≤ 2	67,4	456	32,6	221	5.95^*^
	> 2	74,7	263	25,3	89	
Failed pregnancy	Yes	72,3	149	27,7	57	0.73 ns
	No	69,3	570	30,7	253	
Delivery in a health facility	Yes	30,5	292	69,5	666	0.82 ns
	No	25,4	18	74,6	53	

***: P<0.001;

**: P<0.01;

*: P<0.05; ns: not significant

**Table 2:** Variables influencing the PNC according to the multiple logistic regression models

**Variables**	**Modalities**	**OR**	**(IC 95%)**	
Woman’s origin	Rural	0.75	0.55	1.03
	[Urban]	-	-	-
Woman’s education level	Illiterate	1.29	0,84	1,99
	Primary	1.40	0.98	2.00
	[Secondary and more]	-	-	-
Spouse’s education level	Illiterate	1.67^*^	1.02	2.71
	Primary	1.70^*^	1.21	2.39
	[Secondary and more]	-	-	-
Woman’s employment	Active	1.99^*^	1.23	3.23
	[Inactive]	-	-	-
Spouse’s SPC	SPC1	0.53^*^	0.36	0.78
	[SPC2]	-	-	-
Parity	≤2	1.21	0.88	1.65
	[>2]	-	-	-

**[]:** Reference modality;

*: P<0.05; O.R: Odds Ratio; CI: confidence interval

Indeed, woman’s origin, education level, women’s and spouse’s employment and parity influences the PNC use.

Women of rural origin attended less PNC than those of urban origin. In addition, participants and spouses with a secondary or high education level attended more the PNC.

Similarly, professionally active women and those with spouses belonging to the SPC2 (state functionaries or had liberal professions...) attended more PNC than those who were jobless or whose spouses belong to the SPC1 (workers, craftsmen, employees, drivers, shopkeepers, etc.).

In addition, women with less than two children attended more PNC than those with more than two children. The other variables: failed pregnancies and deliveries in assisted facilities were not associated with the PNC use. Through multivariate analysis, spouse’s education level and couple’s employment were the only variables that determine independently the recourse to the PNC.

## Discussion

This study is one of the very few ones conducted in Marrakesh studying PNC within the population (8). Only, 30.1% of the surveyed women attended PNC. This result confirms the problem we posed at the beginning of the study, about the low use of the post-natal care and the existence of barriers that hinder these medical services.

The frequencies of the exam provided during the PNC indicate a mediocre quality of this consultation because many exams are not systematically provided, although it is an opportunity to detect any anomalies and prevent complications. The majority of the health professionals confirm the lack of continuous training in PNC and the work overload (6, 8).

Besides, 87% of the enrolled women ignore the existence and the importance of the PNC. The use of health services by women and in particular, PNC is a complex phenomenon, influenced by several factors. The socio-psychological characteristics such as knowledge of the PNC and health perception influence their use.

The recourse to the maternal health services and specifically to PNC does not appear as a major concern for these women, despite the possible complications during the postpartum period. This situation can be explained by the lack of knowledge and the importance of the PNC by the studied women (87%) causing the delay or even non-use of these medical care. This is the main reason that hinders them from using these services. This factor has been reported by several studies as a significant barrier to PNC use (9, 10).

Furthermore, disease perception, suggesting an absence of complications (68.6%), is another major reason why women do not use PNC. These findings are similar to the results of some studies in developing countries (11, 12).

Furthermore, the quality of the patient-provider relationship determines the PNC use. This factor was cited by 42% of women; as an obstacle to using PNC. This result is recorded in a similar study (13). Personnel health behaviors is an important determinant of the health services utilization. It includes listening, empathy, time allotted to patient, the care of women in their physical, psychological, and socio-cultural whole and the caregiver integrity.

Similarly, socio-demographic characteristics such as origin, age, education level and women and spouse’s occupation were associated with the PNC use.

Young age, single mother, low education level, occupation of women, the rural origin of women and lower socioeconomic status are important determinants associated with the non-use of the PNC (9, 14– 16).

In our study, these factors also have a very significant association with the recourse to the PNC, except for age and marital status of women. In Morocco, retreat from marriage has moved from 17.3 yr old in 1960 to 26.6 yr old in 2010, can explain partly this finding (17). Second, childbirth is socially conceivable only in the context of marriage.

In addition, low parity (<2) was significantly associated with the PNC use. During the first births, woman takes more care of herself. This has been shown with a group of women in Senegal (18).

Previous experiences such as prenatal care and place of birth are very influential factors on the use of the PNC (12, 14, 15, 19). In our study, the majority of women attended ANC and gave birth in healthcare facility. This did not show a significant association with the use of the PNC.

Limitations of our study are related to the population of studied women; they were enrolled in the public health centers without considering women who have given birth at home and those attending a private sector. This is justified by the constraints of time and limited resources have not allowed us to expand our research.

## Conclusion

This study confirmed the low rate of the PNC and concluded that various determinants explain this fact. The fight against illiteracy, improving household living standards, sensitization of women on the importance of postpartum care, awareness and capacity building of health professionals in the PNC and communication, and the development of a system of home visits for non-users of postnatal care allow improving the PNC rate.

## Ethical considerations

Ethical issues (Including plagiarism, informed consent, misconduct, data fabrication and/or falsification, double publication and/or submission, redundancy, etc.) have been completely observed by the authors.
